# Metagenomic analysis of gut microbiota in colorectal adenocarcinoma in the MENA region

**DOI:** 10.3389/fcimb.2025.1634631

**Published:** 2025-11-17

**Authors:** Mohammad T. Al Bataineh, Nihar Ranjan Dash, Mohamed Mysara, Omnia Saeed, Noura Alkhayyal, Iman M. Talaat, Riyad Bendardaf, Maha Saber-Ayad

**Affiliations:** 1 Department of Basic Medical Sciences, College of Medicine, Yarmouk University, Irbid, Jordan; 2 Clinical Sciences Department, College of Medicine, University of Sharjah, Sharjah, United Arab Emirates; 3 Research Institute for Medical and Health Sciences, University of Sharjah, Sharjah, United Arab Emirates; 4 Bioinformatics Group, Center for Informatics Sciences (CIS), School of Information Technology and Computer Science (ITCS), Nile University, Giza, Egypt; 5 Oncology Department, University Hospital Sharjah, Sharjah, United Arab Emirates; 6 Pathology Department, Faculty of Medicine, Alexandria University, Alexandria, Egypt; 7 Pharmacology Department, Faculty of Medicine, Cairo University, Cairo, Egypt

**Keywords:** adenocarcinoma, colorectal cancer, dysbiosis, microbiota, metagenomics

## Abstract

**Background:**

Growing evidence suggests that gut microbiota plays a role in the development of colorectal cancer (CRC), and a few bacterial strains have been linked to carcinogenesis. Contrary to the Western population, the relationship between pro-cancer microorganisms and CRC among Middle Eastern individuals remains largely unexplored. Ninety-eight samples from Middle Eastern individuals with and without CRC were subjected to microbial profiling based on the 16S rRNA gene.

**Results:**

The CRC group exhibited a more complex gut microbiota with clusters that were significantly distinct from those of the control group. The taxonomic orders Caulobacterales, Rhizobiales, Sphingomonadales, and Burkholderiales, along with the genera Recibecterium and Sphingobium, were overrepresented in the CRC samples based on differential abundance testing between the CRC and control groups. Utilizing 16S-based functional prediction, we identified a significant enrichment of pathways vital for pentose and glucuronate interconversions, metabolism of terpenoids and polyketides, spliceosome, and dTMP kinase pathways within the CRC group. Moreover, we observed a link between Herbaspirillum huttiense and the pathways regulating the actin cytoskeleton; this intriguing connection may provide insights into the molecular mechanisms underlying cytoskeletal rearrangement and carcinogenesis triggered by H. huttiense.

**Conclusions:**

The findings of this study support the connection between gut microbiota and the development of CRC and highlight region-specific microbial signatures that may serve as non-invasive diagnostic biomarkers or predictive tools for early screening in Middle Eastern populations, where CRC is increasingly diagnosed at advanced stages. These insights could inform the development of microbiome-based screening panels and personalized prevention strategies adapted to the MENA region’s unique genetic, dietary, and environmental profiles.

## Introduction

Gut microbiota is an active ecosystem that is critical to human health and disease. Recently, numerous studies have revealed a clear relationship between colorectal cancer (CRC) and altered gut microbiota or gut dysbiosis (Gagnière et al., 2016; Jahani-Sherafat et al., 2018; Wong and Yu, 2019). Although the exact etiology of CRC remains unknown, several factors, including certain types of diets (rich in processed and red meat, low in fiber, and high in fat), smoking, obesity, physical inactivity, low socioeconomic status, age, and alcohol consumption, have been identified as increasing the risk of CRC for an individual (Abarenkov et al., 2010; Doubeni et al., 2012; Al Bataineh et al., 2022). Interestingly, these modifiable environmental factors significantly alter the gut microbiota composition (Watson and Collins, 2011). Emerging evidence suggests a noticeable decrease in intestinal bacterial diversity and richness among CRC patients compared to healthy individuals (Ahn et al., 2013; Saffarian et al., 2019). In addition, CRC patients showed significant alterations in specific bacterial species, such as increased Fusobacterium nucleatum, Porphyromonas, Bacteroides fragilis, Campylobacter, Peptostreptococcus, Enterococcus fecalis, Escherichia coli, and Streptococcus gallolyticus, and a decrease in Faecalibacterium, Blautia, Clostridia, Roseburia, and Bifidobacterium (Wang et al., 2012; Ahn et al., 2013). These changes have resulted in an enrichment of pro-inflammatory opportunistic pathogens and reduced butyrate-producing bacteria. Thus, creating an imbalance in intestinal homeostasis (dysbiosis) could ultimately lead to tumor formation (Feng et al., 2015). Furthermore, the variation in microbiota composition between colorectal cancer patients in the early and advanced stages may potentially point to a direct or indirect function for the gut microbiota in tumor genesis and progression by causing chronic inflammation and creating toxins and metabolites (Sadrekarimi et al., 2022).

The incidence of CRC shows an increasing trend in Middle Eastern Arab countries compared to developed countries, particularly over the last decade (Ferlay et al., 2015). Colorectal cancer (CRC) ranked as the third most frequently diagnosed malignancy among both males and females in the UAE. Non-UAE residents accounted for the majority of CRC cases, representing 71.1%. The disease showed a higher prevalence among males, contributing to 12.5% of all male cancer cases, compared to 7% among females (Shelpai and El-Metwally, 2014).

According to Globocan, in both UAE and Libya, CRC is the 2nd most common cancer in both sexes, whereas it is the seventh in Egypt (W.H. Organization, 2022). Egypt reports a trend of increased early-onset CRC (EOCRC) rates, which was identified more than 25 years ago and has continued over the past few decades (Rashad et al., 2024). While most epidemiological studies have pointed toward factors including lifestyle changes, increasing obesity, and increased consumption of meat, and alteration in food patterns where the regular consumption of traditional foods is being replaced with more Western-style and ready-made foods for this increase in incidence (Arafa and Farhat, 2015; Cox, 2015), no study has explored the composition of the gut microbiome in this population and its alteration in CRC. To investigate the differences in gut microbiota between patients with CRC and controls, we conducted 16S amplicon sequencing on the gut microbiota of Middle Eastern patients diagnosed with CRC and control subjects. Furthermore, we aim to predict the enriched pathways that will illuminate the functional composition of microbial communities using PICRUSt (Phylogenetic Investigation of Communities by Reconstruction of Unobserved States). Our study’s findings support the link between gut microbiota and the development of CRC in a Middle Eastern cohort of patients.

## Materials and methods

### Study population

A retrospective case-control analysis was conducted on CRC specimens collected from the Middle Eastern population, namely from the United Arab Emirates (UAE), Egypt, and Libya. Ninety-eight (51 CRC and 47 non-CRC) formalin-fixed paraffin-embedded (FFPE) tissue blocks were collected from hospitals where patients with CRC underwent tumor resection. For CRC cases, the inclusion criteria were pathologically confirmed CRC following tumor resection. *In situ* carcinomas, benign adenomatous polyps, and malignancies other than carcinomas were excluded from the study. The non-CRC control specimens were obtained from individuals undergoing bowel resection for non-neoplastic and noninflammatory conditions, such as diverticular disease or benign obstruction. Controls were selected after a thorough review of medical records to confirm the absence of colorectal malignancy, inflammatory bowel disease, or other chronic gastrointestinal disorders. Patients who had received antibiotics, probiotics, corticosteroids, or immunosuppressive therapy within three months prior to tissue collection were excluded to minimize potential confounding effects on gut microbiota composition. All FFPEs were anonymized. A histopathologist confirmed the diagnosis and the degree of tumor differentiation (grade), and an oncologist determined the clinical stage following the tumor, node, and metastasis (TNM) staging system (Amin et al., 2017).

### DNA extraction, PCR, sequencing, and sequence processing

Specimens were placed into a MoBio PowerMag Soil DNA Isolation Bead Plate. DNA was extracted following MoBio’s instructions on a KingFisher robot. First, bacterial 16S rRNA genes were PCR-amplified with dual-barcoded primers targeting the V4 region (515F 5’GTGCCAGCMGCCGCGGTAA-3’, and 806R as 5’GGACTACHVGGGTWTCTAAT-3’), per the protocol of Kozich et al. (2013) (Kozich et al., 2013). Next, using Qubit quantification, only the qualified DNA is used to construct a library. For PCR products, the jagged ends of the DNA fragment would be converted into blunt ends using T4 DNA polymerase, Klenow fragment, and T4 Polynucleotide Kinase. Then, add an ‘A’ base to each 3’ end to make it easier to add adapters. Ampure beads would remove fragments that are too short.

### Statistical analysis

Non-parametric analysis was conducted using the Mann–Whitney U test for group comparisons using a pipeline as previously described (Fadrosh et al., 2014; Al Bataineh et al., 2021). All the tests were run at a probability significance level of p < 0.05. All data were analyzed using SPSS software (SPSS Inc., Chicago, IL, USA, version 28).

### Sequencing data processing

Paired-end reads were generated with the Illumina HiSeq/MiSeq platform. The raw data were analyzed using the DADA2 pipeline (V1.24.0, 19) using the tutorial version 1.16. Mostly, the reads were trimmed to lengths of 240 and 160 bps, respectively, for the forward and reverse reads (filter and Trimmed functions). The reads’ error was estimated (LearnErrors function), and poor-quality reads were subsequently removed. Next, paired-end reads with overlap were merged to tags (mergePairs function). Chimeric tags were then identified and removed using the removeBimeraDenovo function, and the tags were binned into amplicon sequence variants (ASVs), up to a single nucleotide difference. Taxonomic ranks were assigned to ASV tags using a native implementation of the naive Bayesian classifier method Classifier (V1.16, 20), using Silva as a reference database (V132, 21) through the assignTaxonomy function. We selected 16S rRNA amplicon sequencing because it minimizes host DNA contamination, captures low-abundant microbial taxa, and provides cost-effective, high-throughput ecological comparisons between groups.

### Diversity analysis

The alpha diversity indices were calculated using Chao and inverse Simpson using the Vegan package (alpha_meas function, V2.6, 22), to reflect species richness and complexity of the community, respectively. Moreover, rarefaction curves were used to explore the species richness of the samples by plotting the number of unique ASV tags observed against the number of reads within each sample. Upon reaching a plateau, these curves suggest reaching a representative bacterial community for the corresponding samples. We applied compositionality-aware methods to assess differentially abundant ASVs and overall microbial community dynamics. For beta-diversity analysis, the permutational multivariate analysis of variance (permanova) approach was applied through vegan package (adonis2 and pairwise.adonis2 functions, 2.6-4, 23) to test the overall differences between the sample and RCM approach (V1.11.4, 24) for visualization, applying central log ratio (CLR) data transformation. This method allows testing whether there are significant differences in beta diversity between groups while controlling for the effect of any pre-specified confounders. To identify the differentially abundant ASV tags, we applied analysis of compositions of microbiomes with bias correction (ancom) method within the ANCOMBC package (ancombc function, V2.0.2, 25), accounting for sampling variations across samples, compositionality, and possible confounders. The differentially abundant ASV tags and higher taxonomical levels were then visualized as a cladogram using LEFSE software (V1.1.2, 26). A significance threshold 0.05 was applied as alpha for both PERMANOVA and ANCOMB models. We considered ‘country’ as a proxy variable that may capture underlying dietary and lifestyle patterns unique to each geographical setting, since these are well-established drivers of microbial composition.

### Functional profiling from 16S data

Gene family abundances from KEGG Orthology (KO) functional space were computed from 16S ASV tags using PICRUSt2 (v2.5.1, 27), and statistical analysis was performed using the statistical analysis of metagenomic profiles (STAMP) software (v2.1.3, 27). We used two groups (Welch t-statistical test) to determine whether the abundance of taxa or functional categories differs significantly between groups of samples. Look for p-values filter > 0.05 and effect size filter < 1.4 to determine whether the differences are statistically significant.

## Results

### Clinical data results

We collected 98 specimens from Middle Eastern individuals with CRC (n=51) and a control group (n=47). The primary demographic information of the subjects in this study includes age, gender, ethnicity, and the grade and TNM stage of CRC cases (**Table 1**). These specimens were obtained from three countries, namely the UAE, Egypt, and Libya (**Table 2**). All CRC cases were confirmed as adenocarcinoma with or without a mucinous component. We further note that while the total cohort size (n = 98) was sufficient for our ecological endpoints, deeper stratification (e.g., by country, type, gender, and age) was inevitable and reduced subgroup sizes. Accordingly, we treated ‘country’ as a cofounder rather than performing multi-way stratified discovery, to ensure stability of the observed CRC vs. Non-CRC effect.

**Table 1:** Demographics of the study cohort.

Characteristics	CRC	Non-CRC
	(n=51)	(n=47)
Age: (mean ± sd, range)	59.8 ± 15.0, 33-86	52.8 ± 11.3, 30-68
Gender (M, F)	31, 20	25, 22
MENA	51	47
Asians	21	30
Africans	30	17
Grade (I, II, III)	10, 37, 4	Not applicable
TNM stage (I, II, III, IV)	9, 15, 19, 8	Not applicable

Baseline parameters of the patient and control groups are matched.

Mann Whitney U test (non-parametric test), p-value = 0.25.

Chi-squared test, Pearson’s Chi square =0.34.

**Table 2:** Distribution of samples across country, disease status (CRC vs non-CRC), sex, and age.

Egypt											
CRC						Non-CRC					
19						9					
Male			Female			Male			Female		
8			11			6			3		
<50	50-75	>75	<50	50-75	>75	<50	50-75	>75	<50	50-75	>75
3	2	3	3	5	3	4	1	1	1	1	1
Libya											
CRC						Non-CRC					
17						0					
Male			Female			Male			Female		
12			5			0			0		
<50	50-75	>75	<50	50-75	>75	<50	50-75	>75	<50	50-75	>75
2	6	3	1	2	1	0	0	0	0	0	0
UAE											
CRC						Non-CRC					
12						30					
Male			Female			Male			Female		
6			6			14			16		
<50	50-75	>75	<50	50-75	>75	<50	50-75	>75	<50	50-75	>75
0	4	2	1	4	1	6	4	4	7	7	2

### Species composition and abundance information

**Figure 1:**
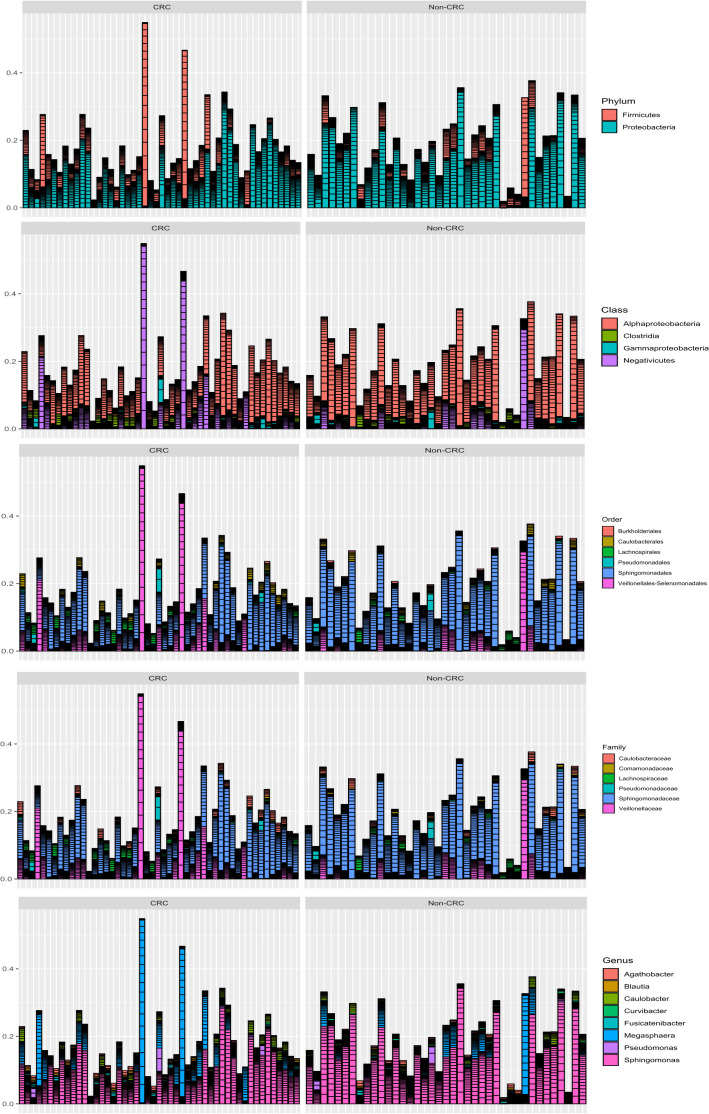
Different taxonomic levels for each sample in CRC and non-CRC. A bar plot generated displays the relative abundance within each sample at various taxonomic levels (namely, phylum, class, order, family, and genus). The x-axis of the plot represents the different samples, while the y-axis represents the relative abundance of taxa within each sample, expressed as a percentage (0-100%).

Deep 16S rRNA amplicon sequencing, combined with the principles of statistical ecology, was used to survey microbiome communities. After pre-processing, using DADA2, the reads within each sample were binned into ASV tags, resulting in a count table for each ASV against the different samples. The average sequencing depth before preprocessing was 43,872; after preprocessing, it reached 15609. Those ASV tags were assigned a taxonomic rank, including phylum, class, order, family, genus, and species in different samples, and were summarized in a histogram, as shown in **Figure 1**.

### Diversity analysis within a single sample

**Figure 2:**
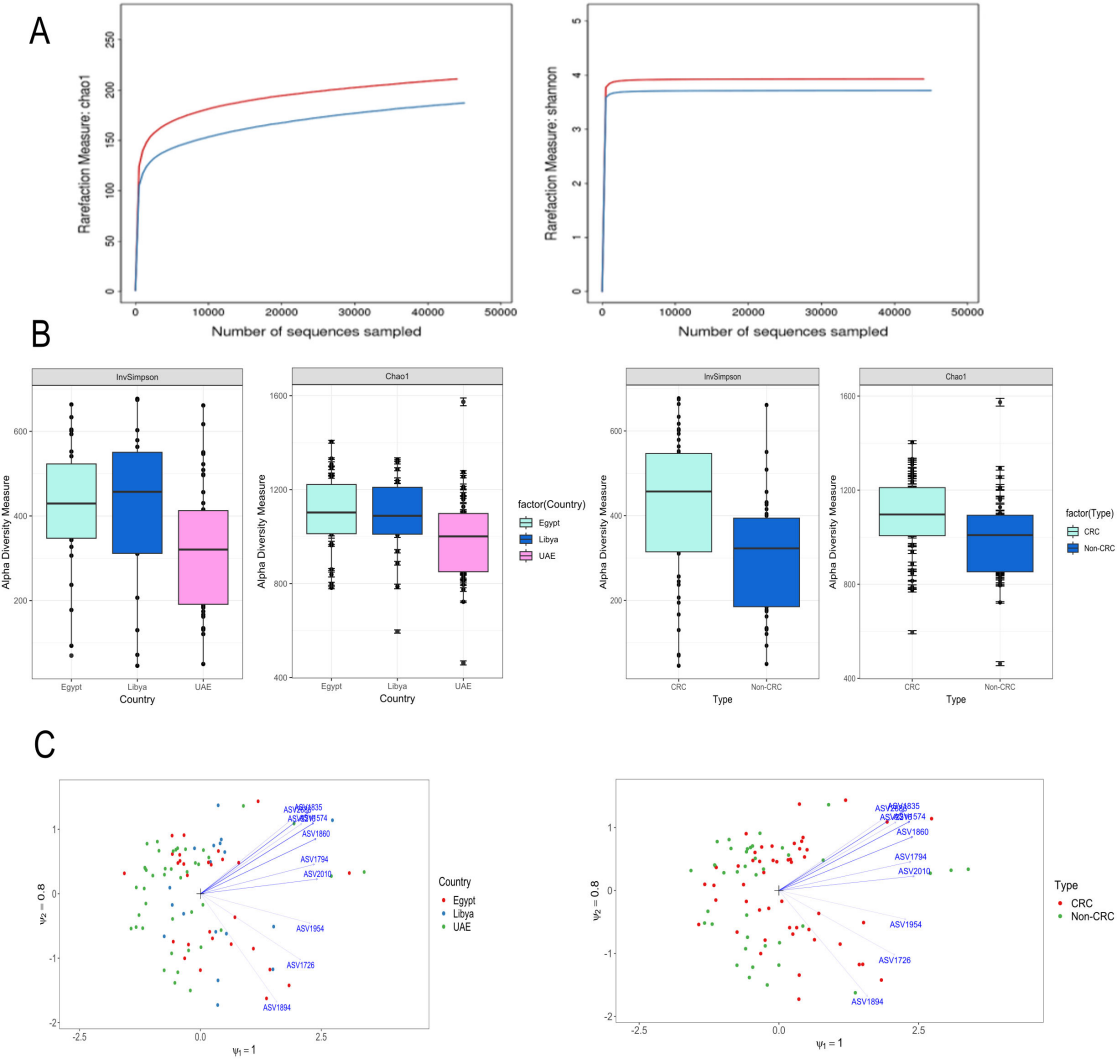
Alpha & Beta-diversity in the samples (n=88). **(A)** Evaluation of the alpha-diversity in the 88 analyzed samples using sample-based rarefaction analysis. The outlined average rarefaction curves were based on the Chao1 and the inverse Simpson index of CRC and healthy samples. **(B)** Boxplots were used to visually display the differences in the alpha diversity (Chao and inverse Simpson) among CRC and non-CRC groups. **(C)** Unconstrained RC(M) method capturing all variability present between the data. Taxa are more abundant than average in samples whose arrows point and less abundant when their arrows point away from the samples.

Diversity analysis within every sample included in the study was applied by calculating alpha diversity to analyze the complexity of species. Briefly, diversity for a sample through several indices, namely, rarefaction curves, Chao1, and Inverse Simpson diversity indices. The rarefaction curves indicated that samples had reached a plateau, indicating adequate sequencing depth (**Figure 2A**). From the results, the CRC samples had a significant increase (p < 0.021) in their bacterial diversity both in terms of richness (Chao) and evenness (inverse Simpson). Moreover, a significant drop in the alpha diversity for the samples originating from the UAE was reported (p < 0.007). In contrast, the samples of Egypt and Libya showed little to no difference in alpha diversity. No differences were noted when assessing gender and age (**Figure 2B** and **Supplementary Figure 1**). This might indicate a possible confounding effect of the country, which we will consider and assess in further analyses.

### Diversity analysis among samples (beta-diversity)

Beta diversity was applied to assess the differences in the microbial communities between different samples (CRC vs. control). After CLR normalization (accounting for compositionality), all potential confounders, namely countries, gender, and age, were assessed through the PERMANOVA models. This method allows testing whether there are significant differences in beta diversity between groups while controlling for the effect of the country. From the results, only the country was found to be a potential confounder when assessed separately or together with the main impacts of the difference between CRC and non-CRC (p-value of 0.007 and 0.037, respectively). On the other hand, no significant difference was reported for gender and age (non-significant p-values of 0.594 and 0.181, respectively). When modeling the main effect separately or together with the country as a confounder, a significant difference was reported (p-value of 0.022 and 0.018, respectively) between the CRC and non-CRC samples. This effect was further visualized through CRM plots for both the country and the main effect (**Figure 2C**).

**Figure 3:**
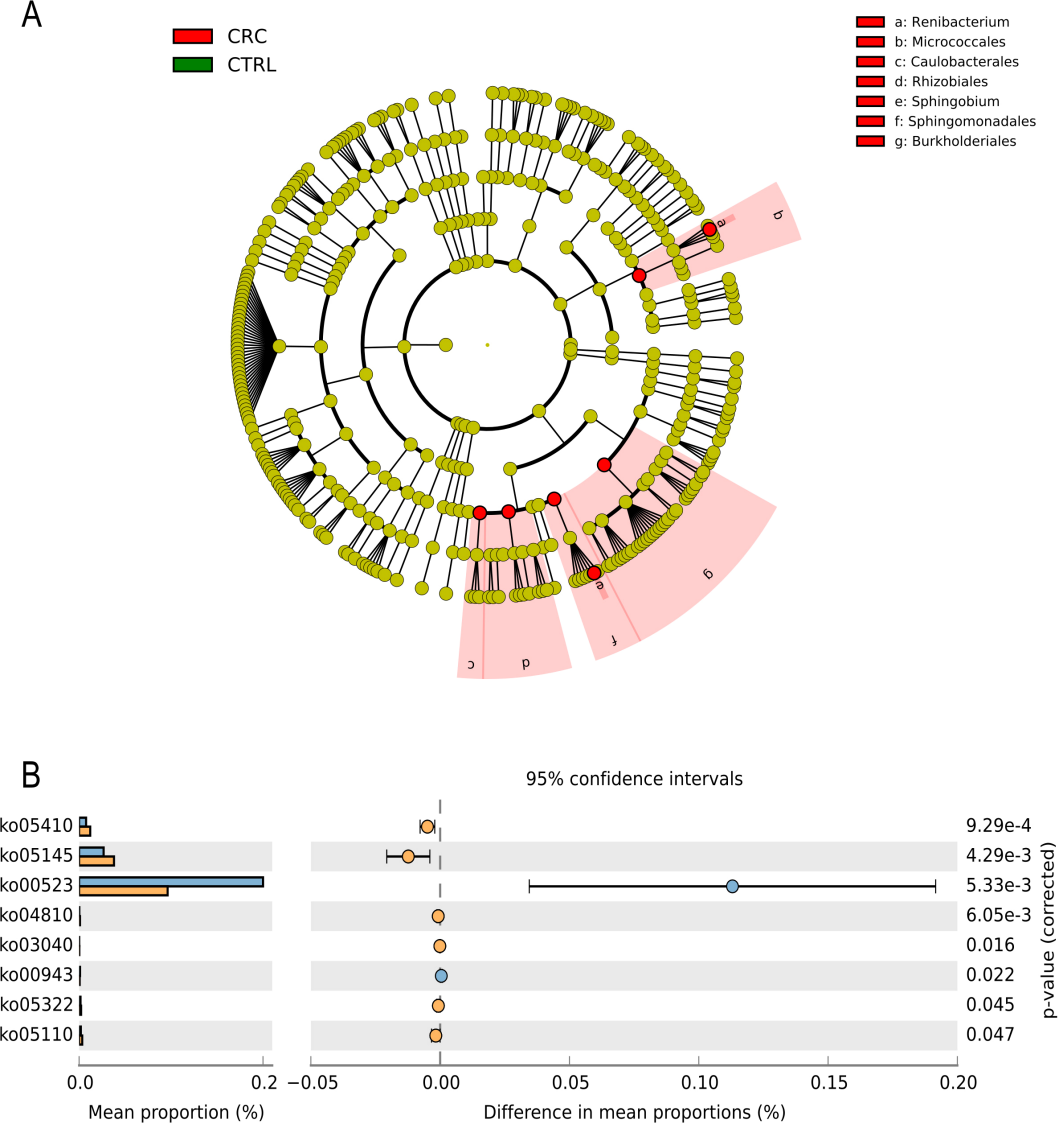
Microbial biomarkers differentially abundant and functional characterization between CRC patients and non-CRC subjects. **(A)** In the Cladogram, red-colored clades indicate the taxonomical groups most represented in CRC vs non-CRC subjects, according to ANCOM-BC results. **(B)** Bar plots of the predicted function expression in both CRC and non-CRC samples using STAMP software.

Microbial differential abundance between CRC patients and non-CRC subjects was tested using ANCOM-BC to account for both the main effect (CRC vs. non-CRC) and the confounding effect (the country of origin). From the results, we have reported an over-representation of several taxonomical orders in the CRC samples, including Caulobacterales, Rhizobiales, Sphingomonadales, and Burkholderiales, as well as Recibacterium and Sphingobium genera (**Figure 3A**). Regarding the member’s differentially abundant because of the main effect, 175 ASVs were more abundant in the CRC samples, while 80 were more abundant in the non-CRC samples (**Table 3**). When applying a 0.05% mean cut-off across all samples, only 24 ASVs remained, representing the more prevalent members capable of inducing a biological effect. In particular, Streptococcus pyogenes, Parapedobacter composti, Bifidobacterium dentium, Herbaspirillum huttiense, and Wujia chipingensis were over-represented in the CRC samples. In contrast, several members of Methylorubrum spp., Brevundimonas spp., Olivibacter spp., and Methylobacterium spp. were identified to be underrepresented in the CRC samples (**Table 3** and **Supplementary Table 1**).

**Table 3:** Differentially abundance samples detected by ANCOM-BC.

Taxonomy	Blast	Coefficient Value	T- Test	CRC Coefficient Direction	Adjusted p-values	Identity Percent	Name of ASVs
Streptococcus	Streptococcus pyogenes	-0.82	-2.03	+	0.00	100.00%	ASV872 ASV987
		-1.14	-2.92		0.000		
Parapedobacter	Parapedobacter composti	-0.21	-0.69	+	0.000	99.2%	ASV1281
Methylobacterium -Methylorubrum	M.populi, M.thiocyanatum, M.rhodesianum, M.extorquens, M.pseudosasae, M.zatmanii, M.suomiense, M.aminovorans	0.61	1.64	–	0.000	>99.6%	ASV632
Bifidobacterium	Bifidobacterium dentium	-1.63	-3.53	+	0.000	99.6%	ASV786
Herbaspirillum	Herbaspirillum huttiense	1.87	4.28	–	0.008	100%	ASV175 ASV258 ASV329 ASV343 ASV368 ASV376 ASV378 ASV410 ASV434 ASV508 ASV628
		2.03	4.68		0.005		
		1.75	3.99		0.013		
		1.97	4.57		0.005		
		1.87	4.19		0.009		
		1.78	4.17		0.009		
		1.81	4.29		0.008		
		1.82	4.32		0.008		
		1.46	3.48		0.069		
		1.72	3.99		0.013		
		1.43	3.72		0.036		
Brevundimonas	B.goettingensis B.huaxiensis B.humi B.albigilva B.staleyi B.poindexterae B.vesicularis B.intermedia B.bacteroides B.aurantiaca B.nasdae	0.70	2.46	–	0.000	100%	ASV1165 ASV1245
		0.40	1.26				
ruminantium group	Wujia chipingensis	-1.21	-2.45	+	0.000	100%	ASV875
Olivibacter	Olivibacter jilunii, Olivibacter oleidegradans	-0.37	-0.79	+	0.000	> 99.2%	ASV904
Acinetobacter	Acinetobacter lwoffii, Prolinoborus fasciculus	-0.09	-0.29	+	0.000	100%	ASV1054
Methylobacterium -Methylorubrum	M.bullatum, M.goesingense, M.adhaesivum, M.gossipiicola, M.iners, M.marchantiae	0.25	0.57	–	0.000	100%	ASV1091

The table reported the ASV tags, their taxonomic classification and their closest hit in NCBI (with their percent identity). Additionally, Coefficients, t-statistics, p-value and the direction of difference were also reported.

### Functional profiling of CRC microbiomes using PICRUST analysis

Using PICRUSt2, it was possible to extrapolate the functional capabilities of those differentially abundant taxa. From the results as in (**Figure 3B**), several pathways were observed to be over-represented in the CRC groups, such as pentose and glucuronate interconversions, toxoplasmosis, regulation of actin cytoskeleton, metabolism of terpenoids and polyketides, spliceosome, dTMP kinase, and vibrio cholerae infection, and two pathways of diseases, namely; hypertrophic cardiomyopathy (genetic cardiac disease) and systemic lupus erythematosus (autoimmune disease). These pathways were primarily driven by the differentially abundant ASVs identified. This was most pronounced in the over-representation of hypertrophic cardiomyopathy, actin cytoskeleton regulation strongly driven by Herbaspirillum huttiense (more detailed in **Supplementary Table 1**).

## Discussion

The current study describes for the first time the make-up, complexity, and functional role of the gut microbiota in Middle Eastern populations concerning colorectal cancer. We found that more complex cancer specimens had a higher level of gut microbiota with varied compositions. A significant functional contribution to signaling networks was identified as being involved in cancer. The changes in the gut microbiota composition associated with CRC in human subjects are a rapidly developing area of research (Sobhani et al., 2011; Ahn et al., 2013). Although the causality is not fully understood, compositional alteration in the gut microbial community and its role in mucosal inflammatory responses are universally reported (Chen et al., 2012; Kostic et al., 2013), especially in Western cohorts. Previous studies have linked the dysregulation of gut microbiota to CRC. For example, studies have linked Fusobacterium nucleatum with cancer as a pro-carcinogenic bacterium in CRC (Lynch and Pedersen, 2016; Liu et al., 2018). This is the first report on gut microbial dysbiosis and its association with CRC among Middle Eastern populations.

### Microbial composition

Changes in the bacterial composition of the gut microbiota have been reported in colorectal cancer, suggesting a significant role of dysbiosis in colorectal carcinogenesis (Gagnière et al., 2016). The higher level of gut microbiota complexity among the CRC group reported in our study may indicate the potential overgrowth of the pro-carcinogenic bacteria. To evaluate species complexity between CRC and control samples in more detail, we assessed the beta diversity after adjusting for possible confounders such as country, gender, and age. We determined a significant separation between the CRC and control groups. The unified approach to unconstrained and constrained visualization of microbiome read count data showed that most of the samples were clustered into two groups, CRC or control, underscoring an exciting difference in bacterial composition between patients with CRC and control individuals.

Next, considering both the confounding factor (the country of origin) and the main effect (CRC vs. non-CRC), we conducted differential abundance testing using ANCOM-BC. Our findings show that the taxonomic orders Caulobacterales, Rhizobiales, Sphingomonadales, and Burkholderiales, as well as the genera Recibacterium and Sphingobium, are overrepresented in the CRC samples. Only 24 ASVs were left after applying a 0.05% mean cut-off to all samples, representing the members most capable of producing a biological effect. The CRC samples had an excess of several bacteria, including Streptococcus pyogenes, Parapedobacter composti, Bifidobacterium dentium, Herbaspirillum huttiense, and Wujia chipingensis. While it was found that some Methylobacterium, Brevundimonas, Olivibacter, and Methylobacterium species were underrepresented in the CRC samples. Furthermore, this approach enables visualization of the discovered biomarkers on taxonomic trees as an effective means for summarizing the results biologically meaningfully while accounting for the confounding effect of the country of origin. This statistically and visually captures the hierarchical relationships inherent in 16S-based taxonomies/phylogenies or ontologies of pathways and biomolecular functions (Golub et al., 1999).

We discovered several important taxa among the CRC group. While Firmicutes and Bacteroidetes constitute the two most important phyla in the gut microbial ecosystem, several bacteria, such as Fusobacterium nucleatum, Escherichia coli, Bacteroides fragilis, Enterococcus faecalis, and Peptostreptococcus stomatis, are known to be increased in CRC patients (Faith et al., 2013; Gagnière et al., 2016). These alterations in the gut microbiota or dysbiosis, are attributed to inducing chronic inflammation, altered immune responses, metabolic pathways, stem cell dynamics, and biosynthesis of harmful metabolites that ultimately affect the host homeostasis, resulting in the pathogenic process, including tumorigenesis (Holmes et al., 2011).

### Functional implications

Functional prediction of the CRC-attributed microbiome revealed the involvement of several genes attributed to several key pathways that may play a role in carcinogenesis, cancer cell proliferation, and metabolism. For example, the present study results showed pentose and glucuronate interconversions, metabolism of terpenoids and polyketides, spliceosome, and dTMP kinase pathways were significantly enriched among the CRC group. These pathways play a pivotal role in controlling intestinal homeostasis (Grazioso et al., 2019). Our results were consistent with previous studies. For example, a pan-cancer analysis of transcriptional metabolic dysregulation in the cancer genome identified a significant dysregulation in these pathways (Rosario et al., 2018). Interestingly, other studies have shed light on the prognostic value of dTMP kinase pathways and emerging roles of the spliceosome in cancer and immunity in cancer cells (Lan et al., 2022; Yang et al., 2022).

We also discovered a connection between hypertrophic cardiomyopathy and the pathways that control the actin cytoskeleton, especially in Herbaspirillum huttiense and other bacteria like Streptococcus pyogenes and Brevundimonas. Through dysregulated production of cytoskeletal proteins such as fibroblast growth factor, fascin-1, radixin, abelson interactor gene-2, and profilin-2, previous research has demonstrated evidence that the actin-cytoskeleton pathway plays a crucial role in inflammatory bowel disease and colorectal cancer (Tsai et al., 2007; Kanaan et al., 2010). H. huttiense is a rare gram-negative bacillus previously reported as an opportunistic pathogen in immunocompromised patients with pneumonia, cirrhosis, and cancer, such as non-small and small-cell lung cancer (Regunath et al., 2015; Liu et al., 2019). Our PICRUSt2 analysis suggests that Herbaspirillum huttiense may play a role in regulating the actin cytoskeleton in CRC samples. However, this association remains speculative, as 16S-based functional profiling may not accurately reflect *in vivo* metabolic activity. Previous research has linked the dysregulation of actin proteins, such as fascin-1 and radixin, to CRC progression. The increased presence of H. huttiense and its production of short-chain fatty acids hint at a potential influence on tumor cell dynamics (Tsai et al., 2007; Regunath et al., 2015; Knox et al., 2022). Further validation through targeted metabolomic analyses or *in vitro* models is necessary to confirm these findings.

Several mechanisms may be implicated; e.g., shift of the host cell metabolism toward non-glycolytic sugar processing, activating the pentose phosphate pathway, increasing NADPH production, which is a key factor in redox balance and nucleotide biosynthesis, supporting tumor growth, and drug metabolism and detoxification of carcinogens via the H. huttiense effect on glucouronidation (Schiliro and Firestein, 2021). This intriguing linkage may provide the basis for the molecular mechanisms of H. huttiense carcinogenesis.

Terpenoids are secondary metabolites produced by microbes, with antibiotic, cytotoxic, or immunomodulatory features (Gozari et al., 2021). The terpenoids, produced by H. huttiense, may interfere with host transcription and epigenetics, modulate the tumor microenvironment, e.g., via immune suppression or inflammation, or lead to metabolic rewiring in tumor cells to adapt to stress or promote proliferation. On the contrary, some studies revealed an anticancer effect of terpenoids (El-Baba et al., 2021). Furthermore, the spliceosome is involved in mRNA splicing, thus regulating gene expression and controlling isoform diversity (Black et al., 2023). In the presence of H. Huttiense, tumor cells may undergo “transcriptomic stress”, leading to alternative splicing and isoform switching of oncogenes and tumor suppressors. In addition, aberrant splicing may be enhanced by microbial metabolites (e.g., lipopolysaccharides or short RNAs). A shift in splicing may enable tumor immune evasion, drug resistance, or metastasis. Noteworthy, alternative splicing is a key regulator in T-cell response (Yong et al., 2025).

Our enrichment results suggest enhanced nucleotide biosynthesis, potentially due to: enhanced tumor proliferation, driven by microbial signals, a remarkable response to DNA damage stress (possibly microbial-derived), and induction of DNA repair and replication programs by H. huttiense-associated inflammation or reactive oxygen species (Escriva et al., 2025).

Previously, researchers discovered that the FadA adhesion protein allows Fusobacterium nucleatum to interact with and invade the colonic epithelium. FadA binds to E-cadherin and Annexin A1, forming a complex that increases β-catenin signaling activity and, as a result, the overexpression of its transcription factors, oncogenes, and inflammatory genes (Rubinstein et al., 2013).

Our results suggest that microbiota can act at several levels; they can directly bind to host adhesion receptors that alter junctions/signaling, secrete effectors/toxins that may modify Rho GTPases or actin, and induce host inflammatory signaling, in addition to other downstream pathways (e.g., NF-κB, Wnt) that secondarily reprogram cytoskeletal regulators (Rubinstein et al., 2013; Xu et al., 2021). The metabolic pathways (likely represented by dTMP kinase, as revealed by PICRUSt analysis) represent another mechanism that can link the composition of the microbiome to cancer metastasis. Previous studies reported that F. nucleatum upregulates CARD3 and enhances autophagy, thus promoting metastasis. Intracellular bacteria commonly manipulate actin/endomembrane trafficking to invade or survive. This can alter host cytoskeleton architecture and signaling in ways that can enhance dissemination (Faoro et al., 2019). In addition, certain microbiota have been linked to alteration in the tumor immune micro-environment, e.g., Tumor-derived CCL20 activated by F. nucleatum not only increases CRC metastasis, but also participates in the reprograming of the tumor immune microenvironment via several mechanisms including M2 macrophage polarization (Xu et al., 2021). Such pathways are novel regarding their link to the microbiome in CRC.

Noteworthy, there is strong association between microbiota and the CRC carcinogenesis, and some causal evidence provided by *in-vivo* studies and experimental models (bacterial mono-colonization, fecal transfers, mutational signatures). Several bacterial virulence factors (adhesins, toxins, genotoxins) are linked to oncogenic effects. Many cohort and case-control studies demonstrated a reproducible enrichment of particular taxa in CRC tissue and stool (notably Fusobacterium nucleatum, pks+ Escherichia coli, and enterotoxigenic Bacteroides fragilis). These observational associations are established across datasets but with no proven causation (Kostic et al., 2013). Antibiotic-treated mice colonized with CRC-associated bacteria or with feces from CRC patients develop more tumors than controls. On the other hand, deletion of specific bacterial virulence genes (e.g., pks island or fadA) reduces tumorigenesis, representing a strong causal relationship in experimental systems (Arthur et al., 2012).

It is well-established that cytoskeleton modifications are linked to cancer cell invasion and metastasis, e.g., epithelial-mesenchymal transition (EMT) is coupled to actin reorganization in a manner to enhance motility and invasion (Yamaguchi and Condeelis, 2007). Actin remodeling (mainly regulated by Rho family GTPases) controls cell polarity, focal adhesions, and formation of invadopodia; the key steps for local invasion and intravasation. Such remodeling ensures the survival of the migrating cells in the circulation on their journey to the metastatic niche (Li and Wang, 2020).

In this study, the identified bacteria among CRC patients suggest an essential role in promoting carcinogenesis and modifying the tumor-immune microenvironment (Fadrosh et al., 2014). We consider the relative sample size as the main limitation of our study. The study’s cohort of 98 participants enables overall comparisons between CRC and non-CRC groups. However, stratification by country, age, or TNM stage results in smaller subgroups, potentially limiting statistical power and the ability to detect subtle microbial differences. Follow-up studies will expand this pilot into a larger cohort to validate our findings and explore specific microbial signatures. As a next step, we will further validate our findings across other populations with different ethnicities and geography, with a balanced number of cases and controls per country.

We also recognize that multi-way stratification (e.g., combining country with stage or age) produces very small strata, limiting interaction testing. While our adjustment models minimized this effect, future studies with larger, balanced cohorts across MENA countries will be needed to validate these patterns.

We acknowledge that while the overall cohort size (n = 98) was adequate for assessing broad ecological differences between CRC and non-CRC groups, stratification by country (UAE, Egypt, and Libya) resulted in smaller subgroup sizes that could limit statistical power for detecting subtle regional variations. To address this, “country” was incorporated as a covariate in PERMANOVA and ANCOM-BC models rather than applying multi-way stratified analysis. This adjustment allowed us to account for regional dietary and lifestyle influences while maintaining the robustness of the CRC versus non-CRC comparisons. However, we recognize that some country-specific microbial signatures might remain undetected due to these constraints. Therefore, our findings should be interpreted as region-level trends that warrant validation in larger, balanced cohorts across the MENA region.

To strengthen and validate the observed microbial associations, we plan to expand this work into a multicenter, prospective cohort across several MENA countries, including the UAE, Egypt, Saudi Arabia, and Jordan, recruiting approximately 300 participants with balanced CRC and non-CRC groups. Future analyses will incorporate shotgun metagenomic sequencing, targeted metabolomics, and host immune transcriptomics to achieve more detailed taxonomic and functional insights. This large-scale validation will facilitate the development of region-specific microbial biomarkers and functional signatures for use in clinical screening and personalized risk assessment programs.

As our discovery dataset was built from FFPE colorectal tissue across the UAE, Egypt, and Libya, enabling region-specific signal detection, but not yet constituting a screening workflow; clinical translation will therefore pivot to stool-based, non-invasive panels derived from the most informative taxa and functions identified here (e.g., Herbaspirillum huttiense, Bifidobacterium dentium, Streptococcus pyogenes, Parapedobacter composti, Sphingobium spp.; pathways in pentose/glucuronate interconversions, terpenoid/polyketide metabolism, spliceosome, and actin-cytoskeleton regulation). These candidates will undergo prospective, multicenter validation at MENA sites using harmonized preanalytics and sequencing/assay pipelines, with primary endpoints of AUC, sensitivity at ≥90% specificity, and added value over FIT/FOBT for colonoscopy triage.

In conclusion, this study offers the first comprehensive characterization of gut microbiota associated with colorectal cancer in Middle Eastern populations. We identified a unique microbial signature characterized by the increased presence of Herbaspirillum huttiense, Bifidobacterium dentium, Streptococcus pyogenes, Parapedobacter composti, and Sphingobium spp. in CRC samples. Functionally, these taxa were connected to the enrichment of pathways involved in pentose and glucuronate interconversions, terpenoid and polyketide metabolism, spliceosome activity, and actin cytoskeleton regulation, processes known to influence tumor growth, immune response, and cytoskeletal dynamics. Overall, these findings suggest that gut microbial imbalance plays a role in CRC development through metabolic and structural changes in host-microbe interactions. Importantly, the identified taxa and pathways could serve as region-specific biomarkers and mechanistic targets for early detection and personalized prevention strategies in the MENA region.

Our findings represent an initial step toward clinical application. The translational aspect includes improving CRC screening in the MENA region through population-customized risk stratification, which can be achieved by combining microbiome data with fecal immunochemical testing or immunohistochemistry of early-detected lesions. Microbiome testing can serve as a predictive or prognostic biomarker for therapy. Also, probiotics, dietary interventions, or even narrow-spectrum antimicrobials can be used if a strong causal relation is established, using a larger sample size and a systematic protocol that verifies the findings and omits confounding factors. Previous protocols have been published to guide the use of microbiome-derived biomarkers for early CRC detection (Ou et al., 2021; Wu et al., 2021; Zwezerijnen-Jiwa et al., 2023). Further large-scale investigations on the microbiome signature of CRC in the MENA region are warranted to help with earlier detection and better management of the disease.

## Data Availability

The original contributions presented in the study are included in the article/**Supplementary Material**. Further inquiries can be directed to the corresponding authors.
