# Supplementary material for: Metagenomic analysis of gut microbiota in colorectal adenocarcinoma in the MENA region
Source: Front Cell Infect Microbiol. 2025 Nov 17;15:1634631. doi: 10.3389/fcimb.2025.1634631 (PMC12665689; doi:10.3389/fcimb.2025.1634631)
Supplement: Supplementary file 1 [file DataSheet1.pdf]

Supplementry Figure 1

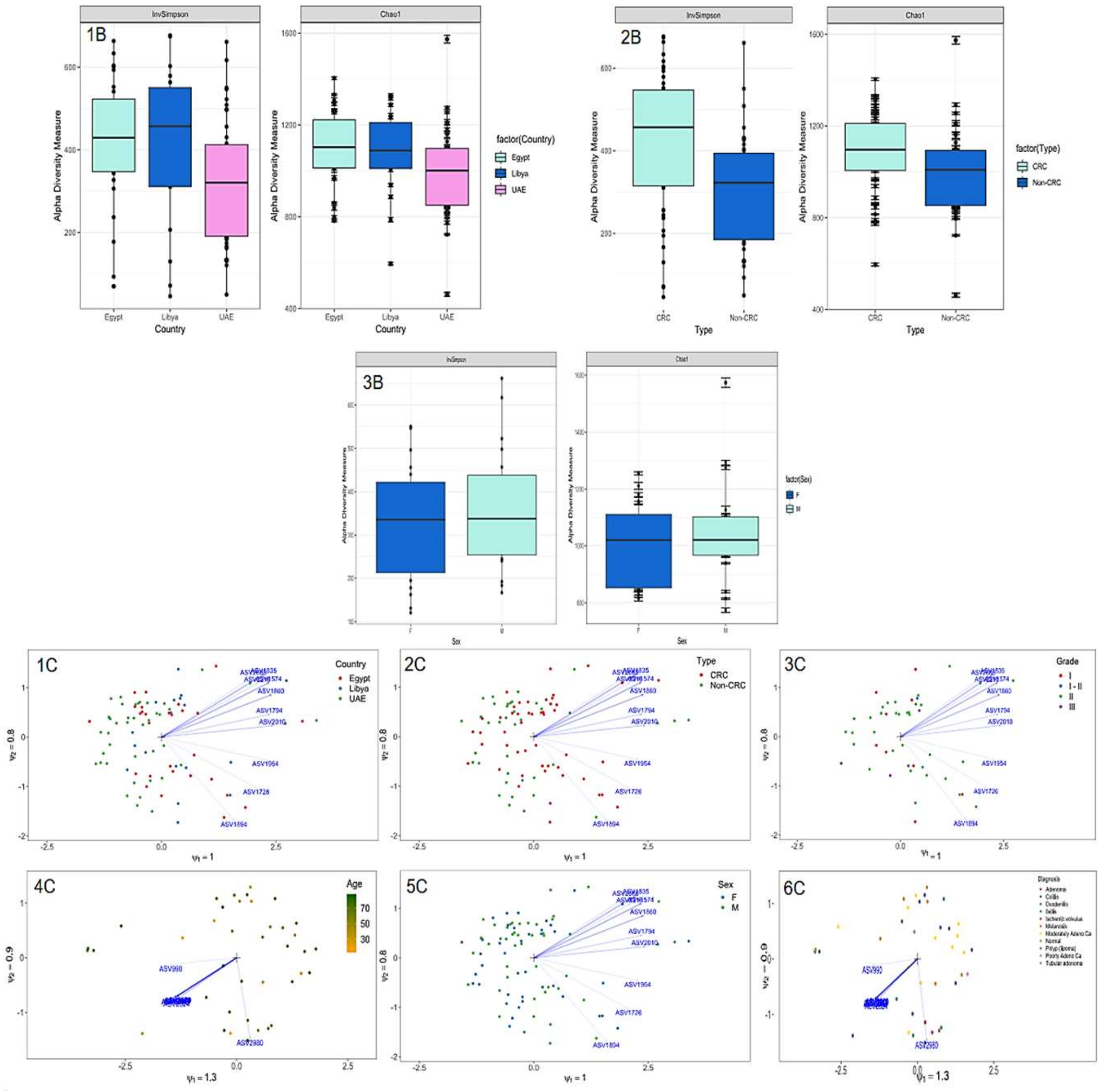

Supplementry Figure 1. Alpha & Beta-diversity in the samples (n=88).
(1-3B)Boxplots were used to visually display the differences in the alpha diversity (Chao and inverse Simpson) among CRC and non-CRC groups.
(1-6C) unconstrained RC(M) method capturing all variability present between the data. Taxa are more abundant than average in samples to which their arrows point and less abundant when their arrows point away from the samples.
